# Sex-disparate safety profile of Obinutuzumab: a pharmacovigilance analysis using the FDA adverse event reporting system

**DOI:** 10.1016/j.clinsp.2026.100865

**Published:** 2026-02-02

**Authors:** Zhixun Bai, Jiaxi Chen, Yongping Lan, Houze Li, Rubin Zheng, Miao Deng, Wenyi Pang

**Affiliations:** a Department of Nephrology, People’s Hospital of Qianxinan Prefecture, Xingyi, Guizhou, China; b Clinical College, Zunyi Medical University, Zunyi, Guizhou, China

**Keywords:** Obinutuzumab, FAERS, Sex differences, Drug safety, Hematologic neoplasms

## Abstract

**Introduction:**

Obinutuzumab, a type II anti‐CD20 monoclonal antibody used for treating B-cell hematologic malignancies, improves outcomes and prolongs progression-free survival. However, its use is linked to adverse reactions that vary by gender. This study aims to assess adverse event signals related to Obinutuzumab using FAERS data, thereby providing safety insights for clinical application.

**Methods:**

FAERS data from Q4 2013 to Q4 2024 were processed in R with duplicate reports removed. Drug names were standardized using Medex UIMA 1.3.8, and adverse events were classified according to MedDRA v26.1. The disproportionality analysis was conducted using the Reporting Odds Ratio (ROR) and Proportional Reporting Ratio (PRR) methods to evaluate adverse reaction signals related to Obinutuzumab. A stratified analysis by gender was performed to enhance the accuracy of data interpretation.

**Results:**

A total of 14,450,774 case reports, 70,790,109 drug cases, and 41,401,915 REAC records were collected. The results showed that although the proportion of male patients in overall adverse event reports was higher, female patients exhibited significantly higher ROR and PRR in systemic adverse reactions such as blood and lymphatic system disorders, suggesting that women may face greater risks. At the Preferred Term (PT) level, strong signals were observed for common adverse events such as myelosuppression and febrile neutropenia. Additionally, abnormal signals for pure red cell aplasia and enteroviral meningitis were prominent only in females.

**Conclusion:**

Obinutuzumab is associated with significant adverse event risks, notably higher in females, highlighting the need for enhanced safety monitoring and further exploration of gender-specific mechanisms in adverse event development.

## Highlights

• Obinutuzumab safety evaluated using FAERS data from 2013–2024.
• Blood and lymphatic disorders show the highest signal strength in both sexes.
• Women show a higher risk of severe hematologic toxicities with Obinutuzumab.
• Novel signals for pure red cell aplasia and enteroviral meningitis in women.

## Introduction

Obinutuzumab is a type II anti-CD20 monoclonal antibody,^1^ primarily used for the treatment of B-cell-related hematologic and lymphatic malignancies, such as non-Hodgkin lymphoma and chronic lymphocytic leukemia.^2^^,^^3^ In clinical trials, Obinutuzumab, when used in combination with other targeted therapies, typically demonstrates a significant improvement in treatment outcomes and progression-free survival. For example, the combination of Venetoclax and Obinutuzumab has been shown to significantly improve survival rates in previously untreated chronic lymphocytic leukemia patients.^4^

Obinutuzumab was first approved by the FDA in 2013 for the treatment of chronic lymphocytic leukemia and later received approval for other types of non-Hodgkin lymphoma. Currently, Obinutuzumab has become a key drug in the treatment regimens for many hematologic and lymphatic malignancies.^5^, ^6^, ^7^ However, the use of Obinutuzumab appears to be associated with a higher risk of adverse reactions. A randomized, controlled phase 3 trial in chronic lymphocytic leukemia showed that the incidence of adverse events was higher in the Obinutuzumab combination therapy group.^8^ Furthermore, multiple studies have similarly indicated that while adding Obinutuzumab improves efficacy, it also increases the risk of adverse events.^5^^,^^9^^,^^10^

The FDA Adverse Event Reporting System (FAERS) is one of the largest drug safety databases in the world, used to collect and analyze Adverse Drug Events (ADEs) related to drug use.^11^ This database provides an opportunity to explore the potential ADEs of Obinutuzumab. This study aims to retrospectively review the data on Obinutuzumab from the FAERS database between the fourth quarter of 2013 and the fourth quarter of 2024, evaluating the occurrence of drug-related adverse events to promote the rational use of clinical medications.

## Methods

### *Data source and preprocessing*

Considering the market launch of Obinutuzumab, this study downloaded the American Standard Code for Information Interchange (ASCII) report files from the FAERS database for the period from the fourth quarter of 2013 to the fourth quarter of 2024,^12^ and processed the data using the R language. Drug names were standardized using Medex UIMA 1.3.8,^13^ and adverse event classification was performed using MedDRA version 26.1.^14^ Duplicate reports were removed, and in cases with the same CASEID, only the report with the most recent date was retained.^15^

### *Data extraction and analysis*

**Fig. 1:**
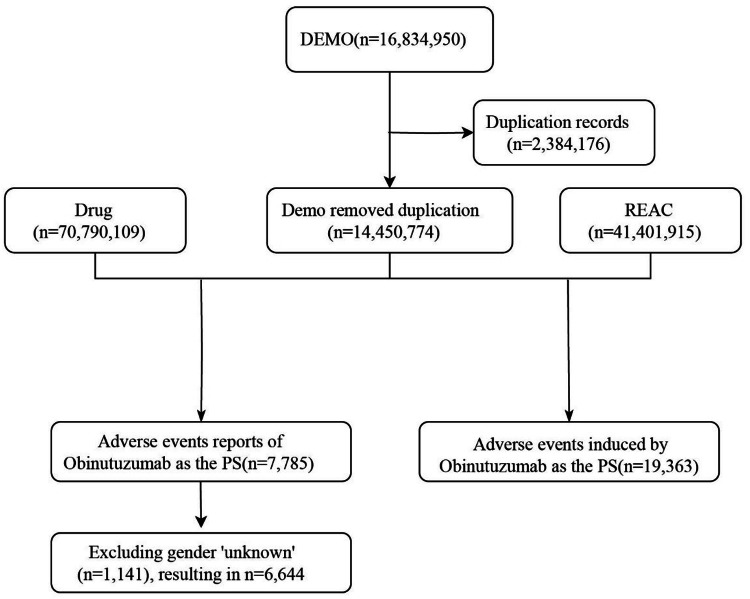
The flow diagram of selecting Obinutuzumab-related ADEs from FAERS database.

Reports related to Obinutuzumab as the primary drug associated with Adverse Drug Events (ADEs) were extracted, covering information such as the report date, patient age and gender, reporter, and region. After preprocessing, this study obtained 14,450,774 report records, 70,790,109 drug cases, and 41,401,915 REAC records (Fig. 1).

This study used the Reporting Odds Ratios (ROR) and Proportional Reporting Ratios (PRR) from disproportionality analysis to assess Adverse Drug Event (ADE) signals. To ensure the comprehensiveness and accuracy of the results, the study stratified by gender, separately evaluating the ROR and PRR values for male and female patients. The advantage of ROR lies in its ability to correct for biases caused by low report volumes of certain events. Compared to ROR, the advantage of PRR is its higher specificity. This study employed both ROR and PRR methods to leverage their respective strengths, aiming to detect more reliable safety signals.

### *Statistical analysis*

The ROR and PRR are based on Table 1, where a ≥ 3, ROR 95% CI (lower limit) > 1, and PRR 95% CI (lower limit) ≥ 1 are considered as positive ADE signals.^16^ The higher the ROR and PRR values, the stronger the ADE signal. The drug retrieval information for this study is provided in Supplementary 1.

$$ROR=\frac{ad}{bc}$$

$$95\%\;CI=e^{\ln\left(\text{ROR}\right)\pm 1.96\sqrt{\left(\frac{1}{a}+\frac{1}{b}+\frac{1}{c}+\frac{1}{d}\right)}}$$

$$PRR=\;\frac{a/\left(a+b\right)}{c/\left(c+d\right)}$$

$$95\%\;CI=e^{\ln\left(\text{PRR}\right)\pm 1.96\sqrt{\left(\frac{1}{a}-\frac{1}{a+b}+\frac{1}{c}-\frac{1}{c+d}\right)}}$$

**Table 1:** Four grid table.

	**Obinutuzumab-related ADEs**	**Non-Obinutuzumab-related ADEs**	**Total**
**Obinutuzumab**	a	b	a + b
**Non-Obinutuzumab**	c	d	c + d
**Total**	a + c	b + d	N = a + b + c + d

Data analysis was conducted using R (version 4.4.2), and the flowchart was created using draw.io software. This study was conducted following the STROBE guidelines.

## Results

### *Basic characteristics of Obinutuzumab-related ADEs*

In the adverse event reports related to Obinutuzumab, the number of male patients (59.23%) was significantly higher than that of female patients (40.77%). Among the reports with clear age data, the most common adverse events were reported in patients over 45-years-old, with fewer reports from those under 45. The majority of reports (67.99%) were from physicians, which increased the reliability of the ADEs information. Reports came from several countries and regions, including the United States (25.86%), Japan (15.58%), China (9.87%), France (7.10%), and Australia (2.97%). In terms of clinical outcomes, hospitalization was the most common (43.19%), followed by other serious outcomes (37.80%) and death (12.32%). Detailed information can be found in Table 2.

**Table 2:** Basic information on ADEs related to Obinutuzumab from the FAERS database.

**Factors**	**Total events (%)**	**Female (%)**	**Male (%)**
Year			
2013	8 (0.12)	4 (50)	4 (50)
2014	162 (2.44)	71 (43.83)	91 (56.17)
2015	293 (4.41)	129 (44.03)	164 (55.97)
2016	536 (8.07)	215 (40.11)	321 (59.89)
2017	607 (9.14)	221 (36.41)	386 (63.59)
2018	561 (8.44)	201 (35.83)	360 (64.17)
2019	458 (6.89)	188 (41.05)	270 (58.95)
2020	576 (8.67)	213 (36.98)	363 (63.02)
2021	455 (6.85)	195 (42.86)	260 (57.14)
2022	822 (12.37)	328 (39.9)	494 (60.1)
2023	859 (12.93)	361 (42.03)	498 (57.97)
2024	1307 (19.67)	583 (44.61)	724 (55.39)
2013‒2024	6644	2709 (40.77)	3935 (59.23)
Age (year)			
< 18	20 (0.30)	7 (35)	13 (65)
18‒45	399 (6.01)	212 (53.13)	187 (46.87)
45‒65	1799 (27.08)	754 (41.91)	1045 (58.09)
≥ 65	3149 (47.40)	1236(39.25)	1913 (60.75)
Unknow	1277 (19.22)	500(39.15)	777 (60.85)
Weight (kg)	70.00 (60.00, 83.00)	62.00 (54.00, 71.00)	77.00 (67.30, 88.00)
Reporter			
Physician	4517 (67.99)	1873 (41.47)	2644 (58.53)
Pharmacist	1329 (20.00)	527 (39.65)	802 (60.35)
Consumer	397 (5.98)	173 (43.58)	224 (56.42)
Other health-professional	382 (5.75)	130 (34.03)	252 (65.97)
Unknown	19 (0.29)	6 (31.58)	13 (68.42)
Reported countries			
United states	1718 (25.86)	661 (38.47)	1057 (61.53)
Japan	1035 (15.58)	472 (45.6)	563 (54.4)
Other	849 (12.78)	355 (41.81)	494 (58.19)
China	656 (9.87)	298 (45.43)	358 (54.57)
France	472 (7.10)	190 (40.25)	282 (59.75)
Germany	424 (6.38)	144 (33.96)	280 (66.04)
Italy	306 (4.61)	125 (40.85)	181 (59.15)
United Kingdom	227 (3.42)	97 (42.73)	130 (57.27)
Australia	197 (2.97)	90 (45.69)	107 (54.31)
Russia	117 (1.76)	50 (42.74)	67 (57.26)
Canada	108 (1.63)	31 (28.7)	77 (71.3)
Spain	101 (1.52)	40 (39.6)	61 (60.4)
Poland	90 (1.35)	42 (46.67)	48 (53.33)
Brazil	89 (1.34)	32(35.96)	57 (64.04)
Israel	88 (1.32)	27 (30.68)	61 (69.32)
Switzerland	61 (0.92)	13 (21.31)	48 (78.69)
Greece	54 (0.81)	18 (33.33)	36 (66.67)
Korea, south	52 (0.78)	24 (46.15)	28 (53.85)
Route			
Intravenous	4377 (65.88)	1769 (40.42)	2608 (59.58)
Other	1367 (20.57)	544 (39.8)	823 (60.2)
Intravenous drip	900 (13.55)	396 (44)	504 (56)
Outcomes			
Hospitalization	2790 (43.19)	1109 (39.75)	1681 (60.25)
Other serious	2442 (37.80)	1015 (41.56)	1427 (58.44)
Death	796 (12.32)	274 (34.42)	522 (65.58)
Life threatening	341 (5.28)	129 (37.83)	212 (62.17)
Disability	72 (1.11)	30 (41.67)	42 (58.33)
Required intervention to prevent permanent impairment/damage	18 (0.28)	4 (22.22)	14 (77.78)
Congenital anomaly	1 (100.00)		1 (100)
Adverse event occurrence time (days)			
< 7	1544 (33.54)	639 (41.39)	905 (58.61)
7‒28	506 (10.99)	229 (45.26)	277 (54.74)
28‒60	297 (6.45)	122 (41.08)	175 (58.92)
≥ 60	1318 (28.63)	503 (38.16)	815 (61.84)
Unknow	939 (20.40)	386 (41.11)	553 (58.89)

### *Obinutuzumab signal mining*

This study found that adverse reactions associated with Obinutuzumab involved 23 System Organ Classes (SOCs). The results showed that eight systems had both ROR 95% CI (lower limit) > 1 and PRR 95% CI (lower limit) ≥ 1. The most common and strongest signal was observed in blood and lymphatic system disorders [N_male_ = 956, ROR_male_ = 5.31 (95% CI: 4.97‒5.68), PRR_male_ = 4.89 (95% CI: 4.61‒5.19); N_female_ = 765, ROR_female_ = 8.62 (95% CI: 7.99‒9.29), PRR_female_ = 7.77 (95% CI: 7.33-8.24)]. The next most significant systems were infections and infestations [N_male_ = 1316, ROR_male_ = 2.56 (95% CI: 2.42‒2.71), PRR_male_ = 2.35 (95% CI: 2.22‒2.49); N_female_ = 911, ROR_female_ = 2.53 (95% CI: 2.36‒2.71), PRR_female_ 2.32 (95%CI: 2.19‒2.46)) and immune system disorders (N_male_ = 214, ROR_male_ = 2.27 (95% CI: 1.98‒2.60), PRR_male_ = 2.24 (95% CI: 1.95‒2.57); N_female_ = 155, ROR_female_ = 1.69 (95% CI: 1.44‒1.98), PRR_female_ = 1.67 (95% CI: 1.43‒1.95)] (Table 3).

**Table 3:** The signal strength of ADEs of Obinutuzumab at the SOC level in FAERS database.

**System organ class**	**Male case reports**	**Male ROR (95% CI)**	**Male PRR (95% CI)**	**Female case reports**	**Female ROR (95% CI)**	**Female PRR (95% CI)**
Blood and lymphatic system disorders	956	5.31 (4.97, 5.68)	4.89 (4.61, 5.19)	765	8.62 (7.99, 9.29)	7.77 (7.33, 8.24)
Infections and infestations	1316	2.56 (2.42, 2.71)	2.35 (2.22, 2.49)	911	2.53 (2.36, 2.71)	2.32 (2.19, 2.46)
Immune system disorders	214	2.27 (1.98, 2.6)	2.24 (1.95, 2.57)	155	1.69 (1.44, 1.98)	1.67 (1.43, 1.95)
Investigations	1018	1.63 (1.52, 1.73)	1.56 (1.47, 1.65)	698	1.85 (1.72, 2.01)	1.77 (1.64, 1.91)
Hepatobiliary disorders	154	1.57 (1.34, 1.84)	1.56 (1.33, 1.82)	107	2.04 (1.69, 2.47)	2.03(1.67, 2.47)
Vascular disorders	381	1.71 (1.55, 1.9)	1.69 (1.53, 1.86)	204	1.55 (1.34, 1.78)	1.53 (1.33, 1.75)
Cardiac disorders	421	1.48 (1.34, 1.63)	1.46 (1.32, 1.61)	211	1.6 (1.39, 1.83)	1.58(1.38, 1.81)
Respiratory, thoracic and mediastinal disorders	729	1.51 (1.4, 1.63)	1.47 (1.36, 1.59)	493	1.49 (1.36, 1.64)	1.46 (1.35, 1.58)
Metabolism and nutrition disorders	242	0.98 (0.87, 1.12)	0.98 (0.87, 1.1)	140	1.03 (0.87, 1.22)	1.03 (0.88, 1.2)
Neoplasms benign, malignant and unspecified (incl cysts and polyps)	362	0.97 (0.87, 1.08)	0.97 (0.88, 1.07)	176	1.11 (0.96, 1.29)	1.11 (0.97, 1.27)
General disorders and administration site conditions	1483	0.83 (0.79, 0.88)	0.86 (0.83, 0.89)	1034	0.75 (0.71, 0.81)	0.79 (0.74, 0.84)
Gastrointestinal disorders	584	0.71 (0.65, 0.77)	0.73 (0.67, 0.79)	479	0.74 (0.67, 0.81)	0.76 (0.7, 0.82)
Injury, poisoning and procedural complications	709	0.65 (0.61, 0.71)	0.68 (0.63, 0.74)	566	0.73 (0.67, 0.8)	0.75 (0.69, 0.81)
Renal and urinary disorders	154	0.61 (0.52, 0.72)	0.62 (0.53, 0.73)	64	0.61 (0.48, 0.79)	0.62 (0.49, 0.78)
Skin and subcutaneous tissue disorders	305	0.61 (0.54, 0.68)	0.62 (0.55, 0.7)	256	0.56 (0.49, 0.63)	0.57 (0.51, 0.64)
Nervous system disorders	430	0.52 (0.47, 0.57)	0.54 (0.49, 0.6)	362	0.59 (0.53, 0.65)	0.61 (0.55, 0.67)
Ear and labyrinth disorders	15	0.36 (0.22, 0.6)	0.36 (0.22, 0.6)	13	0.39 (0.22, 0.66)	0.39 (0.23, 0.68)
Endocrine disorders	9	0.32 (0.17, 0.62)	0.32 (0.17, 0.61)	4	0.22 (0.08, 0.58)	0.22 (0.08, 0.59)
Musculoskeletal and connective tissue disorders	120	0.26 (0.21, 0.31)	0.26 (0.22, 0.31)	135	0.3 (0.25, 0.35)	0.31 (0.26, 0.37)
Eye disorders	58	0.32 (0.25, 0.42)	0.33 (0.26, 0.43)	25	0.16 (0.11, 0.23)	0.16 (0.11, 0.24)
Reproductive system and breast disorders	13	0.18 (0.1, 0.31)	0.18 (0.1, 0.31)	13	0.21 (0.12, 0.36)	0.21 (0.12, 0.36)
Psychiatric disorders	109	0.17 (0.14, 0.2)	0.17 (0.14, 0.21)	67	0.18 (0.14, 0.23)	0.19 (0.15, 0.24)
Congenital, familial and genetic disorders	5	0.17 (0.07, 0.4)	0.17 (0.07, 0.41)			

At the PT level, excluding results with unknown gender, our study identified a total of 147 PTs, with detailed information available in Supplementary 2. Based on the ROR rankings, Table 4 displays the top 30 PTs for different gender groups. The results revealed PTs with high signal strength, such as myelosuppression [N_male_ = 162, ROR = 32.5 (95% CI: 27.78‒38.02), PRR = 31.98 (95% CI: 27.34‒37.41); N_female_=136, ROR = 43.95 (95% CI: 37.05‒52.14), PRR = 43.10 (95% CI: 36.13‒51.41)], pure red cell aplasia [N_female_ = 5, ROR = 23.67 (95% CI: 9.82‒57.06), PRR = 31.98 (95% CI: 9.79‒57.13)], and febrile neutropenia [N_female_ = 123, ROR = 22.97(95% CI: 19.21‒27.47), PRR = 22.58 (95% CI: 18.93‒26.94)]. The most common events included infusion-related reactions, myelosuppression, tumor lysis syndrome, and febrile neutropenia. In addition to the aforementioned results, this study also found that lymphoma transformation, follicular lymphoma, and chronic lymphocytic leukemia recurrence showed high signal strengths in both genders. Notably, although meningitis enteroviral occurred less frequently, its signal strength was the highest and it occurred exclusively in females.

**Table 4:** The top 30 signal strength of adverse events of Obinutuzumab ranked by ROR at the PTs level in FAERS database.

**Soc**	**Pt**	**Male case reports**	**Male ROR (95% CI)**	**Male PRR (95% CI)**	**Female case reports**	**Female ROR (95% CI)**	**Female PRR (95% CI)**
Blood and lymphatic system disorders	Myelosuppression	162	32.5 (27.78, 38.02)	31.98 (27.34, 37.41)	136	43.95 (37.05, 52.14)	43.1 (36.13, 51.41)
Blood and lymphatic system disorders	Aplasia pure red cell				5	23.67 (9.82, 57.06)	23.65 (9.79, 57.13)
Blood and lymphatic system disorders	Febrile neutropenia				123	22.97 (19.21, 27.47)	22.58 (18.93, 26.94)
Infections and infestations	Post-acute COVID-19 syndrome	13	69.42 (39.77, 121.2)	69.33 (40.05, 120.02)	16	90.3 (54.93, 148.45)	90.09 (55.19, 147.06)
Infections and infestations	Cytomegalovirus chorioretinitis	22	43.91 (28.71, 67.15)	43.81(28.46, 67.43)	13	128.12 (73.59, 223.05)	127.88 (73.87, 221.38)
Infections and infestations	Cytomegalovirus enterocolitis	8	42.06 (20.82, 85)	42.03 (20.75, 85.11)	7	129.48 (60.83, 275.58)	129.35 (60.23, 277.81)
Infections and infestations	COVID-19 pneumonia	97	29.08 (23.76, 35.59)	28.8 (23.67, 35.04)	44	30.68 (22.78, 41.32)	30.49 (22.72, 40.91)
Infections and infestations	Neutropenic infection	4	34.78 (12.89, 93.81)	34.77 (12.8, 94.48)			
Infections and infestations	Pneumonia haemophilus	3	32.5 (10.35, 102.11)	32.49 (10.42, 101.26)			
Infections and infestations	Campylobacter infection	5	25.99 (10.73, 62.96)	25.98 (10.75, 62.76)			
Infections and infestations	Hepatitis viral	3	24.28 (7.75, 76.04)	24.28 (7.79, 75.68)			
Infections and infestations	Pneumococcal sepsis	3	20.12 (6.44, 62.9)	20.11 (6.45, 62.68)			
Infections and infestations	Haemophilus infection	4	19.84 (7.39, 53.24)	19.83 (7.44, 52.84)			
Infections and infestations	Campylobacter gastroenteritis	3	19.29 (6.17, 60.3)	19.29 (6.19, 60.12)			
Infections and infestations	Meningitis enteroviral				3	501.91 (149.11, 1689.45)	501.69 (148.83, 1691.18)
Infections and infestations	Superinfection bacterial				3	46.69 (14.94, 145.94)	46.67 (14.97, 145.46)
Infections and infestations	Enterocolitis infectious				3	41.83 (13.39, 130.63)	41.81 (13.41, 130.31)
Infections and infestations	Cytomegalovirus infection				35	27.8 (19.91, 38.8)	27.66 (19.82, 38.6)
Infections and infestations	Pneumonia cytomegaloviral				3	25.94 (8.33, 80.8)	25.93 (8.32, 80.82)
Infections and infestations	Cytomegalovirus colitis				4	22.77 (8.51, 60.88)	22.75 (8.54, 60.62)
Neoplasms benign, malignant and unspecified (incl cysts and polyps)	Lymphoma transformation	10	134.23 (70.15, 256.86)	134.1 (70.23, 256.05)	4	191.23 (69.8, 523.93)	191.12 (70.34, 519.31)
Neoplasms benign, malignant and unspecified (incl cysts and polyps)	Follicular lymphoma	21	121.44 (77.73, 189.73)	121.18 (77.21, 190.2)	19	352.06 (219.32, 565.13)	351.09 (219.34, 561.97)
Neoplasms benign, malignant and unspecified (incl cysts and polyps)	Chronic lymphocytic leukaemia recurrent	14	115.44 (66.92, 199.13)	115.28 (66.59, 199.57)	5	170.77 (69.5, 419.57)	170.64 (69.27, 420.38)
Neoplasms benign, malignant and unspecified (incl cysts and polyps)	B-cell lymphoma recurrent	4	33.74 (12.51, 90.97)	33.72 (12.41, 91.62)	5	160.91 (65.58, 394.87)	160.8 (65.27, 396.14)
Neoplasms benign, malignant and unspecified (incl cysts and polyps)	Richter's syndrome	11	41.57 (22.81, 75.74)	41.52 (22.61, 76.23)	4	74.78 (27.75, 201.49)	74.74 (27.51, 203.08)
Neoplasms benign, malignant and unspecified (incl cysts and polyps)	Tumor flare	3	24.42 (7.8, 76.49)	24.42 (7.84, 76.11)			
Neoplasms benign, malignant and unspecified (incl cysts and polyps)	Metastatic squamous cell carcinoma	3	22.36 (7.14, 69.95)	22.35 (7.17, 69.66)			
Investigations	Cytomegalovirus test positive	12	29.99 (16.93, 53.15)	29.96 (16.97, 52.89)	5	39.38 (16.3, 95.12)	39.35 (16.29, 95.06)
Investigations	Procalcitonin increased	5	16.38 (6.78, 39.56)	16.37 (6.78, 39.55)	3	27.13 (8.71, 84.53)	27.12 (8.7, 84.53)
Investigations	Human rhinovirus test positive	3	33.53 (10.67, 105.4)	33.52 (10.75, 104.47)			
Investigations	Blood immunoglobulin g decreased	7	20.25 (9.6, 42.72)	20.24 (9.61, 42.63)			
Investigations	Lymphocyte count decreased	55	17.52 (13.42, 22.88)	17.43 (13.25, 22.93)			
Investigations	Cd4 lymphocytes decreased				9	68.97 (35.63, 133.5)	68.88 (35.37, 134.12)
Immune system disorders	Hypogammaglobulinaemia	21	17.35 (11.28, 26.69)	17.31(11.25, 26.64)	15	29.04 (17.46, 48.31)	28.98 (17.41, 48.24)
Immune system disorders	Secondary immunodeficiency	4	23.48 (8.74, 63.07)	23.47 (8.81, 62.53)			
Immune system disorders	Cytokine release syndrome				36	28.03 (20.18, 38.95)	27.89 (19.99, 38.92)
Respiratory, thoracic and mediastinal disorders	Obliterative bronchiolitis				8	72.2 (35.83, 145.52)	72.12 (35.61, 146.05)
Respiratory, thoracic and mediastinal disorders	Organising pneumonia				10	22.51 (12.08, 41.94)	22.48 (12.01, 42.09)
Metabolism and nutrition disorders	Tumor lysis syndrome	95	43.37 (35.32, 53.24)	42.95 (35.31, 52.25)	44	81.37 (60.28, 109.84)	80.86 (60.26, 108.5)
Metabolism and nutrition disorders	Hyperphosphataemia	8	18.15 (9.04, 36.47)	18.14 (8.96, 36.73)			
General disorders and administration site conditions	Vaccination failure				5	27.21 (11.28, 65.63)	27.19 (11.26, 65.68)
General disorders and administration site conditions	Hyperpyrexia				10	24.88 (13.35, 46.37)	24.85 (13.27, 46.53)
Vascular disorders	Hyperaemia	6	19.57 (8.74, 43.81)	19.56 (8.76, 43.69)			
Skin and subcutaneous tissue disorders	Prurigo				3	45.22 (14.47, 141.31)	45.2 (14.5, 140.88)
Injury, poisoning and procedural complications	Infusion related reaction	266	33.05 (29.22, 37.38)	32.18 (28.61, 36.2)	204	27.11 (23.57, 31.18)	26.34 (22.96, 30.21)

## Discussion

Obinutuzumab is a type II glycoengineered anti-CD20 monoclonal antibody initially developed to overcome resistance to rituximab in B-cell malignancies. Studies have shown that it is superior to rituximab in the treatment of hematologic malignancies, such as B-cell lymphoma.^17^ Obinutuzumab is primarily administered via intravenous infusion, where it specifically binds to the CD20 antigen on the surface of B-cells and kills target cells through multiple mechanisms, such as Antibody-Dependent Cell-Mediated Cytotoxicity (ADCC) and Antibody-Dependent Cellular Phagocytosis (ADCP).^18^^,^^19^ Unlike type I anti-CD20 antibodies (such as Rituximab), Obinutuzumab effectively induces direct cell death but does not target the antigen-antibody complex to lipid rafts, resulting in lower Complement-Dependent Cytotoxicity (CDC).^18^ Additionally, due to the afucosylation of its Fc region and the optimized conformation of its hinge region, Obinutuzumab enhances direct cell death induction and ADCC activity.^18^^,^^20^ In a previous randomized phase II clinical trial (GAGE), the efficacy of monotherapy with Obinutuzumab in treating Chronic Lymphocytic Leukemia (CLL) patients and the dose optimization were investigated. The results demonstrated significant efficacy for Obinutuzumab in untreated CLL patients.^21^ A more recent clinical trial found that combination therapy with Obinutuzumab showed better treatment outcomes in patients with relapsed/refractory chronic lymphocytic leukemia.^22^ Overall, the efficacy of Obinutuzumab in treating B-cell-related malignancies has been well-established. Furthermore, in recent years, the therapeutic effects of Obinutuzumab in autoimmune-related diseases, such as lupus, rheumatoid arthritis, and primary membranous nephropathy, have also gained increasing recognition.^23^^,^^24^ Clinically, the most common adverse reactions to this drug include Infusion-Related Reactions (IRRs) and neutropenia, which are typically associated with severe toxicity.^8^^,^^25^ The use of Obinutuzumab is also expanding, with applications in conditions like lupus nephritis, antiphospholipid syndrome, and ANCA-associated vasculitis.^26^, ^27^, ^28^ However, there is still limited understanding of the adverse reactions associated with Obinutuzumab. With the increasing diversity of its applications, monitoring its real-world usage and adverse events to ensure its safety and efficacy is crucial. This study systematically evaluated the adverse reactions related to Obinutuzumab by deeply mining the FAERS database from the fourth quarter of 2013 to the fourth quarter of 2024, providing valuable safety information and revealing new potential risks. This data supports medical practice and the rational use of the drug.

This study observed that reports of adverse events related to Obinutuzumab were more common in male patients than in female patients. It is noteworthy that in blood and lymphatic system disorders, the ROR value (8.62, 95% CI: 7.99‒9.29) and PRR value (7.77, 95% CI: 7.33‒8.24) for female patients were significantly higher than the ROR value (5.31, 95% CI: 4.97‒5.68) and PRR value (4.89, 95% CI: 4.61‒5.19) for male patients, indicating that females may face a higher risk when using Obinutuzumab. Since the majority of adverse event reports (67.99%) were submitted by physicians, the credibility of the results presented is high.

As a monoclonal antibody, the most common adverse event associated with Obinutuzumab is Infusion-Related Reactions (IRRs), which are linked to the drug's high affinity for FcγRIII. Obinutuzumab tightly binds to CD20, triggering FcγR activation, leading to the recruitment of effector cells and a strong release of cytokines, resulting in clinical symptoms.^25^ Neutropenia and tumor lysis syndrome are also common adverse reactions.^29^^,^^30^ At the PT level, in addition to myelosuppression and febrile neutropenia, this study also identified pure red cell aplasia, which is not mentioned in the drug labeling. Although there were only 5 female patients, the ROR value reached 23.67 (95% CI: 9.82‒57.06) and the PRR value reached 23.65 (95% CI: 9.79‒57.13), indicating the need for further attention and investigation. Additionally, PTs such as post-acute COVID-19 syndrome, cytomegalovirus chorioretinitis, cytomegalovirus enterocolitis, COVID-19 pneumonia, and enteroviral meningitis also showed high signal strength, especially enteroviral meningitis, which had the highest ROR and PRR values, with ROR at 501.91 (95% CI: 149.11‒1689.45) and PRR at 501.69 (95% CI: 148.83‒1691.18), and it occurred exclusively in female patients. A recent case report mentioned two female patients receiving Obinutuzumab treatment who developed disseminated enteroviral infections. One patient tested positive for enterovirus in the cerebrospinal fluid through PCR, and both patients' symptoms improved rapidly after discontinuing Obinutuzumab and administering 0.8 g/kg of intravenous immunoglobulin.^31^ Furthermore, a phase 3 randomized controlled trial found that infections associated with COVID-19 were more severe in the Obinutuzumab group.^10^ A recent report also mentioned the occurrence of cytomegalovirus enterocolitis after Obinutuzumab combined with bendamustine treatment.^32^ These findings are consistent with our conclusions.

This study found that pure red cell aplasia and enteroviral meningitis related to Obinutuzumab occurred exclusively in female patients. The occurrence of pure red cell aplasia in female patients may involve complex interactions between different gender-related immune system regulation, erythropoiesis regulation mechanisms, or cytokine release patterns. Although there is currently insufficient evidence to suggest that Obinutuzumab treatment is more likely to cause pure red cell aplasia in female patients, these findings warrant further clinical research and data analysis to reveal the potential impact of gender differences on drug-related adverse reactions. During Obinutuzumab treatment, a recent case report confirmed the detection of enterovirus in the cerebrospinal fluid of some female patients, and their clinical condition rapidly improved after discontinuing Obinutuzumab and administering IVIg.^31^ Generally, females tend to have stronger immune responses than males, especially in viral infections. Estrogen is believed to promote B-cell activation and antibody production,^33^ which helps combat viruses and other pathogens.^34^^,^^35^ This suggests that the gender differences in the occurrence of enteroviral meningitis may be related to differences in immune responses, hormone levels, and reactions to immunosuppression between females and males. It indicates the potential existence of a gender-specific immune response mechanism, which warrants further investigation. Further research into this phenomenon may help clarify the mechanism behind Obinutuzumab-induced enteroviral infections and provide insights for personalized treatment and immune monitoring. Considering gender differences, developing gender-specific monitoring protocols will help improve treatment efficacy and the precision of personalized management.

It is important to emphasize that the above discussion regarding the adverse reactions of Obinutuzumab and their potential mechanisms remains speculative. The specific causal relationships need to be further verified through research. The occurrence of adverse reactions is influenced by various factors, including drug characteristics, individual differences, and underlying conditions. Therefore, more in-depth experimental and clinical studies are required to better understand the mechanisms behind these reactions. In addition, adverse events such as pure red cell aplasia and enteroviral meningitis, as identified in the study, are relatively rare. Given the rarity of these events, the authors recommend that these signals be further validated in independent cohorts or confirmed through targeted studies. This validation is crucial for determining the causal relationship between these adverse reactions and Obinutuzumab. Meanwhile, clinicians should continue to closely monitor adverse reactions during Obinutuzumab treatment and implement timely intervention measures.

Although this study provides scientific evidence for the safety assessment of Obinutuzumab, there are still some limitations. As the FAERS database is a spontaneous reporting system, its inherent characteristics, such as underreporting and incomplete case information, may affect the results, particularly regarding gender differences. Despite these limitations, the identification and detection of adverse reaction signals for Obinutuzumab in the FAERS database still provide valuable insights for further research and clinical treatment plans. Additionally, the efficacy and safety of Obinutuzumab for different diseases still require ongoing monitoring.

## Conclusion

Obinutuzumab is associated with significant adverse event risks, notably higher in females, highlighting the need for enhanced safety monitoring and further exploration of gender-specific mechanisms in adverse event development.

### Declarations

Ethics approval and consent to participate: Not applicable.

Consent for publication: Not applicable.

### Authors’ contributions

Conceptualization, J.C., Y.L., and Z.B.; methodology, J.C., Z.B., H.L., and R.Z.; validation, J.C., Y.L., and H.L.; writing-original draft preparation, J.C.; data curation, Y.L., H.L., R.Z., M.D., and W.P. All the authors have read and agreed with the published version of the manuscript.

## Funding

This research was funded by the 10.13039/501100001809National Natural Science Foundation of China (grant n° 82260106), the College Students’ Innovation Training Program under project numbers 2024106610965. Qianxinan Prefecture Science and Technology Project (ZHOU KE HE YI XUE 2025-1).

## Data availability statement

The dataset generated and analyzed during and during the current study is available from the corresponding author on reasonable request.

## Declaration of competing interest

The authors declare no conflicts of interest.

## Appendix Supplementary materials

Image, application 1
