# Supplementary material for: Sex-disparate safety profile of Obinutuzumab: a pharmacovigilance analysis using the FDA adverse event reporting system
Source: Clinics (Sao Paulo). 2026 Feb 2;81:100865. doi: 10.1016/j.clinsp.2026.100865 (PMC12886070; doi:10.1016/j.clinsp.2026.100865)
Supplement: Supplementary file 1 [file mmc1.docx]

CLINICS-D-25-00667_Supplementary Material

**Supplementary Table 1**

| **Drugname (11376)** | **prod_ai** | **ADEs number** |
| --- | --- | --- |
| Obinutuzumab. | Obinutuzumab | 4499 |
| Obinutuzumab | Obinutuzumab | 3390 |
| Gazyva | Obinutuzumab | 2201 |
| Gazyvaro | Obinutuzumab | 1011 |
| Ga101 | Obinutuzumab | 167 |
| Obintuzumab | Obinutuzumab | 11 |
| Bendamustine; Obinutuzumab | Bendamustine; Obinutuzumab | 8 |
| Ro5072759 | Obinutuzumab | 7 |
| Obinutuzumab (monoclonal antibodies) (unknown) | Obinutuzumab | 6 |
| Blinded Obinutuzumab | Obinutuzumab | 6 |
| Afutuzumab | Obinutuzumab | 6 |
| Obinutuzumab (liquid) | Obinutuzumab | 5 |
| Gazyva intravenous solution 1000 mg | Obinutuzumab | 5 |
| Obinutuzumab solution for infusion | Obinutuzumab | 4 |
| Ga101 (Obinutuzumab) | Obinutuzumab | 4 |
| Gazyvaro 1000 mg | Obinutuzumab | 3 |
| Ga-101 | Obinutuzumab | 3 |
| Obinutuzumab (Obinutuzumab 25 mg/mL inj, vil, 40 mL) | Obinutuzumab | 2 |
| Obinutuzumab 1000 mg genentech | Obinutuzumab | 2 |
| Obinutuzumab (infusion) | Obinutuzumab | 2 |
| Obinutuzumab (Obinutuzumab) | Obinutuzumab (Obinutuzumab) | 2 |
| Chlorambucil w/Obinutuzumab | Chlorambucil w/ Obinutuzumab | 2 |
| Obinutuzumab 1000mg | Obinutuzumab | 2 |
| Gazyva chemotherapy | Gazyva chemotherapy | 2 |
| Obinutuzumab, 1000 mg | Obinutuzumab | 2 |
| Ga?101 | Obinutuzumab | 2 |
| Gazyvaro 1000 mg, solution a diluer pour perfusion | Obinutuzumab | 2 |
| Gazyva (chlorambucil, Obinutuzumab) | Chlorambucil; Obinutuzumab | 2 |
| Obinutuzumab (genetical recombination) | Obinutuzumab | 2 |
| Obinutuzumab solution for injection | Obinutuzumab | 1 |
| Obinutuzumab monoclonal antibodies | Obinutuzumab | 1 |
| Chlorambucil; Obinutuzumab | Chlorambucil; Obinutuzumab | 1 |
| Gazyva Obinutuzumab | Gazyva Obinutuzumab | 1 |
| Gazyva-Obinutuzumab | Gazyva-Obinutuzumab | 1 |
| Gazyva immunotherapy | Gazyva immunotherapy | 1 |
| Obinatumumab | Obinutuzumab | 1 |
| Obinituzumab | Obinutuzumab | 1 |
| Obinotuzumab | Obinotuzumab | 1 |
| Obinultuzumab | Obinultuzumab | 1 |
| Obinutuzumab (inv) | Obinutuzumab | 1 |
| Obinutuzumab (Obinutuzumab 25 mg/mL inj, vil, 40 mL) | Obinutuzumab | 1 |
| Lenalidomide; Obinutuzumab | Lenalidomide; Obinutuzumab | 1 |
| G?benda [Obinutuzumab] | Obinutuzumab | 1 |
| Obinutuzumab | Obinutuzumab | 1 |
| Gazyva intravenous | Obinutuzumab | 1 |

**Supplementary Table 2**

| **Soc** | **Pt** | **Male case reports** | **Male ROR (95% CI)** | **Male PRR (95% CI)** | **Female case reports** | **Female ROR (95% CI)** | **Female PRR (95% CI)** | |
| --- | --- | --- | --- | --- | --- | --- | --- | --- |
| Infections and infestations | Post-acute COVID-19 syndrome | 13 | 69.42 (39.77, 121.2) | 69.33 (40.05, 120.02) | 16 | 90.3 (54.93, 148.45) | 90.09 (55.19, 147.06) | |
| Infections and infestations | Cytomegalovirus chorioretinitis | 22 | 43.91 (28.71, 67.15) | 43.81 (28.46, 67.43) | 13 | 128.12 (73.59, 223.05) | 127.88 (73.87, 221.38) | |
| Infections and infestations | Cytomegalovirus enterocolitis | 8 | 42.06 (20.82, 85) | 42.03 (20.75, 85.11) | 7 | 129.48 (60.83, 275.58) | 129.35 (60.23, 277.81) | |
| Infections and infestations | COVID-19 pneumonia | 97 | 29.08 (23.76, 35.59) | 28.8(23.67, 35.04) | 44 | 30.68 (22.78, 41.32) | 30.49 (22.72, 40.91) | |
| Infections and infestations | Superinfection bacterial | 3 | 13.67 (4.39, 42.64) | 13.67 (4.39, 42.61) | 3 | 46.69 (14.94, 145.94) | 46.67 (14.97, 145.46) | |
| Infections and infestations | Pneumonia cytomegaloviral | 6 | 13.86 (6.2, 30.97) | 13.85 (6.2, 30.93) | 3 | 25.94 (8.33, 80.8) | 25.93 (8.32, 80.82) | |
| Infections and infestations | Cytomegalovirus infection | 41 | 11.39 (8.37, 15.5) | 11.35 (8.29, 15.53) | 35 | 27.8 (19.91, 38.8) | 27.66 (19.82, 38.6) | |
| Infections and infestations | Progressive multifocal leukoencephalopathy | 21 | 13.75 (8.95, 21.15) | 13.73 (8.92, 21.13) | 16 | 17.4 (10.64, 28.45) | 17.36 (10.64, 28.34) | |
| Infections and infestations | Coronavirus infection | 22 | 13.35 (8.77, 20.33) | 13.32 (8.83, 20.1) | 16 | 17.95 (10.98, 29.36) | 17.92 (10.98, 29.25) | |
| Infections and infestations | Escherichia sepsis | 4 | 6.67 (2.5, 17.81) | 6.67 (2.5, 17.77) | 3 | 12.9 (4.15, 40.1) | 12.9 (4.14, 40.21) | |
| Infections and infestations | Neutropenic sepsis | 10 | 6.88 (3.7, 12.82) | 6.88 (3.67, 12.88) | 6 | 10.63 (4.77, 23.7) | 10.62 (4.75, 23.72) | |
| Infections and infestations | Cytomegalovirus viraemia | 6 | 6 (2.69, 13.38) | 6 (2.69, 13.4) | 4 | 12.06 (4.52, 32.2) | 12.05 (4.52, 32.11) | |
| Infections and infestations | Hepatitis b reactivation | 5 | 6.02 (2.5, 14.51) | 6.02 (2.49, 14.54) | 3 | 10.97 (3.53, 34.09) | 10.97 (3.52, 34.19) | |
| Infections and infestations | COVID-19 | 230 | 6.15 (5.4, 7.01) | 6.03 (5.26, 6.92) | 212 | 7.89 (6.88, 9.05) | 7.68 (6.7, 8.81) | |
| Infections and infestations | Neutropenic infection | 4 | 34.78 (12.89, 93.81) | 34.77 (12.8, 94.48) |  |  |  |  |
| Infections and infestations | Pneumonia haemophilus | 3 | 32.5 (10.35, 102.11) | 32.49 (10.42, 101.26) |  |  |  |  |
| Infections and infestations | Campylobacter infection | 5 | 25.99 (10.73, 62.96) | 25.98 (10.75, 62.76) |  |  |  |  |
| Infections and infestations | Hepatitis viral | 3 | 24.28 (7.75, 76.04) | 24.28 (7.79, 75.68) |  |  |  |  |
| Infections and infestations | Pneumococcal sepsis | 3 | 20.12 (6.44, 62.9) | 20.11 (6.45, 62.68) |  |  |  |  |
| Infections and infestations | Haemophilus infection | 4 | 19.84 (7.39, 53.24) | 19.83 (7.44, 52.84) |  |  |  |  |
| Infections and infestations | Campylobacter gastroenteritis | 3 | 19.29 (6.17, 60.3) | 19.29 (6.19, 60.12) |  |  |  |  |
| Infections and infestations | Febrile infection | 3 | 11.64 (3.74, 36.27) | 11.64 (3.73, 36.28) |  |  |  |  |
| Infections and infestations | Ophthalmic herpes zoster | 3 | 10.54 (3.38, 32.81) | 10.53 (3.38, 32.82) |  |  |  |  |
| Infections and infestations | Opportunistic infection | 3 | 10.26 (3.29, 31.93) | 10.25 (3.29, 31.95) |  |  |  |  |
| Infections and infestations | Pseudomonal sepsis | 3 | 9.74 (3.13, 30.31) | 9.73 (3.12, 30.33) |  |  |  |  |
| Infections and infestations | Pulmonary sepsis | 4 | 9.58 (3.58, 25.62) | 9.58 (3.6, 25.53) |  |  |  |  |
| Infections and infestations | Epididymitis | 4 | 8.13 (3.04, 21.73) | 8.13 (3.05, 21.66) |  |  |  |  |
| Infections and infestations | Bacterial sepsis | 5 | 7.66 (3.18, 18.46) | 7.66 (3.17, 18.5) |  |  |  |  |
| Infections and infestations | Pneumonia viral | 4 | 6.68 (2.5, 17.83) | 6.67 (2.5, 17.77) |  |  |  |  |
| Infections and infestations | Varicella zoster virus infection | 3 | 6.44 (2.07, 20.03) | 6.44 (2.07, 20.07) |  |  |  |  |
| Infections and infestations | Hepatitis e | 3 | 6.03 (1.94, 18.74) | 6.03 (1.93, 18.79) |  |  |  |  |
| Infections and infestations | Abdominal infection | 3 | 5.66 (1.82, 17.58) | 5.65 (1.81, 17.61) |  |  |  |  |
| Infections and infestations | Meningitis enteroviral |  |  |  | 3 | 501.91 (149.11, 1689.45) | 501.69 (148.83, 1691.18) | |
| Infections and infestations | Enterocolitis infectious |  |  |  | 3 | 41.83 (13.39, 130.63) | 41.81 (13.41, 130.31) | |
| Infections and infestations | Cytomegalovirus colitis |  |  |  | 4 | 22.77 (8.51, 60.88) | 22.75 (8.54, 60.62) | |
| Infections and infestations | Cytomegalovirus infection reactivation |  |  |  | 6 | 18.48 (8.28, 41.23) | 18.46 (8.26, 41.23) | |
| Infections and infestations | Pneumocystis jirovecii pneumonia |  |  |  | 7 | 7.22 (3.44, 15.17) | 7.22 (3.43, 15.21) | |
| Infections and infestations | Bronchopulmonary aspergillosis |  |  |  | 3 | 5.89 (1.9, 18.29) | 5.89 (1.89, 18.36) | |
| Infections and infestations | Aspergillus infection |  |  |  | 3 | 5.59 (1.8, 17.34) | 5.58 (1.79, 17.39) | |
| Infections and infestations | Septic shock |  |  |  | 19 | 5.31 (3.38, 8.33) | 5.3 (3.38, 8.32) | |
| Investigations | Cytomegalovirus test positive | 12 | 29.99 (16.93, 53.15) | 29.96 (16.97, 52.89) | 5 | 39.38 (16.3, 95.12) | 39.35 (16.29, 95.06) | |
| Investigations | Procalcitonin increased | 5 | 16.38 (6.78, 39.56) | 16.37 (6.78, 39.55) | 3 | 27.13 (8.71, 84.53) | 27.12 (8.7, 84.53) | |
| Investigations | Blood immunoglobulin g decreased | 7 | 20.25 (9.6, 42.72) | 20.24 (9.61, 42.63) | 5 | 17.16 (7.13, 41.34) | 17.15 (7.1, 41.43) | |
| Investigations | Lymphocyte count decreased | 55 | 17.52 (13.42, 22.88) | 17.43 (13.25, 22.93) | 40 | 16.35 (11.97, 22.33) | 16.26 (11.88, 22.25) | |
| Investigations | Lymphocyte count increased | 10 | 14.84 (7.95, 27.67) | 14.82 (7.92, 27.75) | 3 | 8.95(2.88, 27.81) | 8.95 (2.87, 27.9) | |
| Investigations | Neutrophil count decreased | 69 | 8.88 (7, 11.26) | 8.83 (6.98, 11.17) | 66 | 15.29 (11.99, 19.5) | 15.16 (11.98, 19.18) | |
| Investigations | Neutrophil count abnormal | 4 | 7.64 (2.86, 20.43) | 7.64 (2.87, 20.36) | 3 | 9.76 (3.14, 30.33) | 9.76 (3.13, 30.42) | |
| Investigations | Blood lactate dehydrogenase increased | 20 | 7.82 (5.04, 12.14) | 7.81 (5.07, 12.02) | 10 | 8.84 (4.75, 16.46) | 8.83 (4.72, 16.53) | |
| Investigations | Oxygen saturation decreased | 60 | 5.82 (4.51, 7.51) | 5.79 (4.49, 7.47) | 45 | 7.05 (5.25, 9.45) | 7.01 (5.22, 9.41) | |
| Investigations | Human rhinovirus test positive | 3 | 33.53 (10.67, 105.4) | 33.52 (10.75, 104.47) |  |  |  |  |
| Investigations | Lymphocyte count abnormal | 3 | 14.47 (4.64, 45.13) | 14.47 (4.64, 45.1) |  |  |  |  |
| Investigations | Granulocyte count decreased | 3 | 11.03 (3.54, 34.36) | 11.03 (3.54, 34.38) |  |  |  |  |
| Investigations | Blood phosphorus increased | 4 | 7.14 (2.67, 19.08) | 7.14 (2.68, 19.02) |  |  |  |  |
| Investigations | Transaminases increased | 26 | 6.15 (4.18, 9.04) | 6.13 (4.14, 9.07) |  |  |  |  |
| Investigations | White blood cell count abnormal | 6 | 5.55 (2.49, 12.38) | 5.55 (2.48, 12.4) |  |  |  |  |
| Investigations | Cd4 lymphocytes decreased |  |  |  | 9 | 68.97 (35.63, 133.5) | 68.88 (35.37, 134.12) | |
| Investigations | Eastern cooperative oncology group performance status worsened |  |  |  | 3 | 18.28 (5.88, 56.88) | 18.28 (5.87, 56.97) | |
| Investigations | Drug specific antibody present |  |  |  | 7 | 11.62 (5.53, 24.41) | 11.61 (5.51, 24.45) | |
| Investigations | Platelet count decreased |  |  |  | 86 | 8.23 (6.65, 10.18) | 8.14 (6.56, 10.1) | |
| Investigations | Blood uric acid increased |  |  |  | 3 | 7.33 (2.36, 22.75) | 7.32 (2.35, 22.81) | |
| Investigations | Blood pressure decreased |  |  |  | 40 | 5.97 (4.37, 8.14) | 5.94 (4.34, 8.13) | |
| Investigations | Body temperature increased |  |  |  | 12 | 5.42 (3.07, 9.55) | 5.41 (3.06, 9.55) | |
| Investigations | Breath sounds abnormal |  |  |  | 3 | 5.25 (1.69, 16.29) | 5.25 (1.68, 16.36) | |
| Neoplasms benign, malignant and unspecified (incl cysts and polyps) | Lymphoma transformation | 10 | 134.23 (70.15, 256.86) | 134.1 (70.23, 256.05) | 4 | 191.23 (69.8, 523.93) | 191.12 (70.34, 519.31) | |
| Neoplasms benign, malignant and unspecified (incl cysts and polyps) | Follicular lymphoma | 21 | 121.44 (77.73, 189.73) | 121.18 (77.21, 190.2) | 19 | 352.06 (219.32, 565.13) | 351.09 (219.34, 561.97) | |
| Neoplasms benign, malignant and unspecified (incl cysts and polyps) | Chronic lymphocytic leukaemia recurrent | 14 | 115.44 (66.92, 199.13) | 115.28 (66.59, 199.57) | 5 | 170.77 (69.5, 419.57) | 170.64 (69.27, 420.38) | |
| Neoplasms benign, malignant and unspecified (incl cysts and polyps) | B-cell lymphoma recurrent | 4 | 33.74 (12.51, 90.97) | 33.72 (12.41, 91.62) | 5 | 160.91 (65.58, 394.87) | 160.8 (65.27, 396.14) | |
| Neoplasms benign, malignant and unspecified (incl cysts and polyps) | Richter's syndrome | 11 | 41.57 (22.81, 75.74) | 41.52 (22.61, 76.23) | 4 | 74.78 (27.75, 201.49) | 74.74 (27.51, 203.08) | |
| Neoplasms benign, malignant and unspecified (incl cysts and polyps) | Chronic lymphocytic leukaemia | 13 | 11.28 (6.54, 19.48) | 11.27 (6.51, 19.51) | 5 | 15.91 (6.61, 38.31) | 15.9 (6.58, 38.41) | |
| Neoplasms benign, malignant and unspecified (incl cysts and polyps) | B-cell lymphoma | 8 | 10.31 (5.14, 20.68) | 10.31 (5.19, 20.47) | 3 | 11.43 (3.68, 35.53) | 11.43 (3.67, 35.62) | |
| Neoplasms benign, malignant and unspecified (incl cysts and polyps) | Squamous cell carcinoma | 23 | 10.2 (6.76, 15.38) | 10.18 (6.75, 15.36) | 7 | 9.8 (4.66, 20.58) | 9.79 (4.65, 20.62) | |
| Neoplasms benign, malignant and unspecified (incl cysts and polyps) | Diffuse large b-cell lymphoma | 11 | 6.07 (3.36, 10.98) | 6.07 (3.37, 10.93) | 8 | 12.56 (6.27, 25.17) | 12.55 (6.32, 24.92) | |
| Neoplasms benign, malignant and unspecified (incl cysts and polyps) | Squamous cell carcinoma of skin | 10 | 5.89 (3.17, 10.97) | 5.89 (3.15, 11.03) | 3 | 6.23 (2.01, 19.36) | 6.23 (2, 19.42) | |
| Neoplasms benign, malignant and unspecified (incl cysts and polyps) | Tumor flare | 3 | 24.42 (7.8, 76.49) | 24.42 (7.84, 76.11) |  |  |  |  |
| Neoplasms benign, malignant and unspecified (incl cysts and polyps) | Metastatic squamous cell carcinoma | 3 | 22.36 (7.14, 69.95) | 22.35 (7.17, 69.66) |  |  |  |  |
| Neoplasms benign, malignant and unspecified (incl cysts and polyps) | Mantle cell lymphoma | 4 | 9.7 (3.63, 25.93) | 9.69 (3.64, 25.82) |  |  |  |  |
| Neoplasms benign, malignant and unspecified (incl cysts and polyps) | Basal cell carcinoma | 32 | 9.39 (6.63, 13.31) | 9.37 (6.58, 13.33) |  |  |  |  |
| Neoplasms benign, malignant and unspecified (incl cysts and polyps) | Diffuse large b-cell lymphoma recurrent | 4 | 8.36 (3.13, 22.34) | 8.36 (3.14, 22.27) |  |  |  |  |
| Neoplasms benign, malignant and unspecified (incl cysts and polyps) | Transitional cell carcinoma | 3 | 8 (2.57, 24.9) | 8 (2.57, 24.93) |  |  |  |  |
| Neoplasms benign, malignant and unspecified (incl cysts and polyps) | Adenocarcinoma | 3 | 6.97 (2.24, 21.68) | 6.97 (2.24, 21.72) |  |  |  |  |
| Neoplasms benign, malignant and unspecified (incl cysts and polyps) | Hodgkin's disease | 5 | 5.54 (2.3, 13.34) | 5.54 (2.29, 13.38) |  |  |  |  |
| Neoplasms benign, malignant and unspecified (incl cysts and polyps) | Adenocarcinoma of colon |  |  |  | 3 | 15.3 (4.92, 47.58) | 15.3 (4.91, 47.69) | |
| Neoplasms benign, malignant and unspecified (incl cysts and polyps) | Lung adenocarcinoma |  |  |  | 3 | 10.14 (3.26, 31.5) | 10.14 (3.25, 31.6) | |
| Neoplasms benign, malignant and unspecified (incl cysts and polyps) | Lymphoma |  |  |  | 8 | 5.89 (2.94, 11.8) | 5.89 (2.97, 11.7) | |
| Neoplasms benign, malignant and unspecified (incl cysts and polyps) | Myelodysplastic syndrome |  |  |  | 6 | 5.65 (2.54, 12.59) | 5.65 (2.53, 12.62) | |
| Blood and lymphatic system disorders | Myelosuppression | 162 | 32.5 (27.78, 38.02) | 31.98 (27.34, 37.41) | 136 | 43.95 (37.05, 52.14) | 43.1 (36.13, 51.41) | |
| Blood and lymphatic system disorders | Febrile neutropenia | 167 | 11.96 (10.26, 13.94) | 11.77 (10.06, 13.77) | 123 | 22.97 (19.21, 27.47) | 22.58 (18.93, 26.94) | |
| Blood and lymphatic system disorders | Cytopenia | 30 | 11.79 (8.23, 16.9) | 11.76 (8.26, 16.74) | 23 | 20.66 (13.7, 31.15) | 20.6 (13.65, 31.09) | |
| Blood and lymphatic system disorders | Aplasia pure red cell | 6 | 9.22 (4.13, 20.58) | 9.21 (4.12, 20.57) | 5 | 23.67 (9.82, 57.06) | 23.65 (9.79, 57.13) | |
| Blood and lymphatic system disorders | Neutropenia | 176 | 7.28 (6.27, 8.45) | 7.16 (6.24, 8.21) | 141 | 10.84 (9.17, 12.81) | 10.64 (9.1, 12.45) | |
| Blood and lymphatic system disorders | Agranulocytosis | 22 | 5.96 (3.92, 9.06) | 5.95 (3.94, 8.98) | 14 | 8.15 (4.82, 13.78) | 8.14 (4.8, 13.82) | |
| Blood and lymphatic system disorders | Haematotoxicity | 10 | 6.73 (3.61, 12.53) | 6.72 (3.59, 12.58) | 5 | 6.42 (2.67, 15.43) | 6.41 (2.65, 15.48) | |
| Blood and lymphatic system disorders | Febrile bone marrow aplasia | 6 | 6.97 (3.12, 15.55) | 6.96 (3.12, 15.55) |  |  |  |  |
| Blood and lymphatic system disorders | Lymphocytosis | 3 | 6.88 (2.21, 21.4) | 6.88 (2.21, 21.44) |  |  |  |  |
| Blood and lymphatic system disorders | Disseminated intravascular coagulation |  |  |  | 12 | 12.49 (7.08, 22.03) | 12.47 (7.06, 22.02) | |
| Blood and lymphatic system disorders | Granulocytopenia |  |  |  | 4 | 10.63 (3.98, 28.38) | 10.63 (3.99, 28.32) | |
| Blood and lymphatic system disorders | Thrombocytopenia |  |  |  | 92 | 10.4 (8.46, 12.78) | 10.27 (8.44, 12.49) | |
| Blood and lymphatic system disorders | Haemolysis |  |  |  | 5 | 8.46 (3.52, 20.35) | 8.45 (3.5, 20.41) | |
| Blood and lymphatic system disorders | Bone marrow failure |  |  |  | 16 | 8.06 (4.93, 13.18) | 8.05 (4.93, 13.14) | |
| Blood and lymphatic system disorders | Splenomegaly |  |  |  | 6 | 7.62 (3.42, 16.97) | 7.61 (3.41, 17) | |
| Blood and lymphatic system disorders | Pancytopenia |  |  |  | 37 | 7.52 (5.44, 10.39) | 7.48 (5.47, 10.24) | |
| Blood and lymphatic system disorders | Immune thrombocytopenia |  |  |  | 3 | 6.9 (2.22, 21.42) | 6.9 (2.21, 21.51) | |
| Blood and lymphatic system disorders | Leukopenia |  |  |  | 28 | 5.73 (3.95, 8.31) | 5.71 (3.93, 8.29) | |
| Blood and lymphatic system disorders | Lymphopenia |  |  |  | 8 | 5.53 (2.76, 11.07) | 5.53 (2.78, 10.98) | |
| Respiratory, thoracic and mediastinal disorders | Organising pneumonia | 14 | 12.2 (7.21, 20.65) | 12.18 (7.17, 20.68) | 10 | 22.51 (12.08, 41.94) | 22.48 (12.01, 42.09) | |
| Respiratory, thoracic and mediastinal disorders | Tachypnoea | 23 | 8.03 (5.33, 12.1) | 8.01 (5.31, 12.09) | 11 | 9.16 (5.07, 16.57) | 9.15 (5.08, 16.47) | |
| Respiratory, thoracic and mediastinal disorders | Bronchospasm | 20 | 9.54 (6.14, 14.81) | 9.52 (6.19, 14.65) |  |  |  |  |
| Respiratory, thoracic and mediastinal disorders | Obliterative bronchiolitis |  |  |  | 8 | 72.2 (35.83, 145.52) | 72.12 (35.61, 146.05) | |
| Respiratory, thoracic and mediastinal disorders | Lung infiltration |  |  |  | 8 | 12.77 (6.38, 25.59) | 12.76 (6.43, 25.34) | |
| Respiratory, thoracic and mediastinal disorders | Hypoxia |  |  |  | 23 | 7.09 (4.7, 10.68) | 7.07 (4.68, 10.67) | |
| Respiratory, thoracic and mediastinal disorders | Pneumonitis |  |  |  | 18 | 6.85 (4.31, 10.88) | 6.83 (4.27, 10.93) | |
| Respiratory, thoracic and mediastinal disorders | Interstitial lung disease |  |  |  | 26 | 6.48 (4.41, 9.53) | 6.46 (4.37, 9.56) | |
| Respiratory, thoracic and mediastinal disorders | Acute pulmonary oedema |  |  |  | 3 | 6.45 (2.08, 20.04) | 6.45 (2.07, 20.1) | |
| Respiratory, thoracic and mediastinal disorders | Respiratory distress |  |  |  | 13 | 5.51 (3.2, 9.51) | 5.51 (3.18, 9.54) | |
| General disorders and administration site conditions | Vaccination failure | 4 | 12.89 (4.82, 34.51) | 12.89 (4.84, 34.34) | 5 | 27.21 (11.28, 65.63) | 27.19 (11.26, 65.68) | |
| General disorders and administration site conditions | Temperature intolerance | 17 | 10.58 (6.57, 17.06) | 10.57 (6.6, 16.92) | 22 | 16.13 (10.6, 24.54) | 16.08 (10.65, 24.27) | |
| General disorders and administration site conditions | Chills | 222 | 11.81 (10.34, 13.5) | 11.57 (10.09, 13.27) | 118 | 8.87 (7.39, 10.64) | 8.73 (7.32, 10.41) | |
| General disorders and administration site conditions | Hyperpyrexia | 5 | 6.08 (2.53, 14.64) | 6.08 (2.52, 14.69) | 10 | 24.88 (13.35, 46.37) | 24.85 (13.27, 46.53) | |
| General disorders and administration site conditions | Extravasation | 4 | 7.1 (2.66, 18.96) | 7.09 (2.66, 18.89) |  |  |  |  |
| General disorders and administration site conditions | Chest discomfort | 88 | 6.33 (5.13, 7.81) | 6.28 (5.06, 7.79) |  |  |  |  |
| General disorders and administration site conditions | Physical deconditioning | 3 | 5.47 (1.76, 17.01) | 5.47 (1.76, 17.05) |  |  |  |  |
| General disorders and administration site conditions | Non-cardiac chest pain |  |  |  | 3 | 10.68 (3.44, 33.18) | 10.67 (3.42, 33.26) | |
| General disorders and administration site conditions | Sudden death |  |  |  | 3 | 5.61 (1.81, 17.42) | 5.61 (1.8, 17.49) | |
| General disorders and administration site conditions | Pyrexia |  |  |  | 197 | 5.49 (4.76, 6.32) | 5.36 (4.67, 6.15) | |
| Immune system disorders | Hypogammaglobulinaemia | 21 | 17.35 (11.28, 26.69) | 17.31 (11.25, 26.64) | 15 | 29.04 (17.46, 48.31) | 28.98 (17.41, 48.24) | |
| Immune system disorders | Cytokine release syndrome | 51 | 11.37 (8.63, 14.99) | 11.32 (8.6, 14.89) | 36 | 28.03 (20.18, 38.95) | 27.89 (19.99, 38.92) | |
| Immune system disorders | Anaphylactic shock | 29 | 7.53 (5.22, 10.85) | 7.51 (5.17, 10.9) | 18 | 6.7 (4.22, 10.65) | 6.68 (4.17, 10.69) | |
| Immune system disorders | Secondary immunodeficiency | 4 | 23.48 (8.74, 63.07) | 23.47 (8.81, 62.53) |  |  |  |  |
| Immune system disorders | Anaphylactoid reaction | 5 | 10.59 (4.39, 25.54) | 10.59 (4.38, 25.58) |  |  |  |  |
| Immune system disorders | Serum sickness |  |  |  | 5 | 22.14 (9.18, 53.35) | 22.12 (9.16, 53.44) | |
| Hepatobiliary disorders | Hepatosplenomegaly | 5 | 9.84 (4.08, 23.71) | 9.83 (4.07, 23.75) |  |  |  |  |
| Hepatobiliary disorders | Hepatitis toxic | 3 | 8.52 (2.74, 26.51) | 8.52 (2.73, 26.55) |  |  |  |  |
| Hepatobiliary disorders | Hypertransaminasaemia |  |  |  | 8 | 10.94 (5.46, 21.9) | 10.93 (5.5, 21.7) | |
| Hepatobiliary disorders | Hepatocellular injury |  |  |  | 14 | 7.98 (4.72, 13.49) | 7.97 (4.69, 13.53) | |
| Hepatobiliary disorders | Hepatic cytolysis |  |  |  | 9 | 6.94 (3.61, 13.36) | 6.93 (3.63, 13.23) | |
| Hepatobiliary disorders | Hepatic function abnormal |  |  |  | 16 | 5.47 (3.35, 8.93) | 5.46 (3.34, 8.91) | |
| Metabolism and nutrition disorders | Tumor lysis syndrome | 95 | 43.37 (35.32, 53.24) | 42.95 (35.31, 52.25) | 44 | 81.37 (60.28, 109.84) | 80.86 (60.26, 108.5) | |
| Metabolism and nutrition disorders | Hyperphosphataemia | 8 | 18.15 (9.04, 36.47) | 18.14 (8.96, 36.73) | 3 | 20.87 (6.71, 64.95) | 20.86 (6.69, 65.02) | |
| Metabolism and nutrition disorders | Hyperuricaemia | 6 | 6.4 (2.87, 14.27) | 6.4 (2.87, 14.29) | 3 | 11.48 (3.7, 35.69) | 11.48 (3.68, 35.78) | |
| Vascular disorders | Hyperaemia | 6 | 19.57 (8.74, 43.81) | 19.56 (8.76, 43.69) |  |  |  |  |
| Vascular disorders | Flushing | 63 | 5.91 (4.61, 7.57) | 5.88 (4.56, 7.59) |  |  |  |  |
| Injury, poisoning and procedural complications | Infusion related reaction | 266 | 33.05 (29.22, 37.38) | 32.18 (28.61, 36.2) | 204 | 27.11 (23.57, 31.18) | 26.34 (22.96, 30.21) | |
| Injury, poisoning and procedural complications | Incorrect drug administration rate | 4 | 5.89 (2.2, 15.72) | 5.89 (2.21, 15.69) |  |  |  |  |
| Skin and subcutaneous tissue disorders | Prurigo |  |  |  | 3 | 45.22 (14.47, 141.31) | 45.2 (14.5, 140.88) | |
| Renal and urinary disorders | Cystitis haemorrhagic |  |  |  | 4 | 13.74 (5.15, 36.7) | 13.74 (5.16, 36.61) | |
| Gastrointestinal disorders | Pneumoperitoneum | 3 | 7.13 (2.29, 22.16) | 7.12 (2.28, 22.19) |  |  |  |  |
| Cardiac disorders | Supraventricular tachycardia | 8 | 5.56 (2.77, 11.13) | 5.55 (2.79, 11.02) |  |  |  |  |
